# Inhibition of Virulence Gene Expression in *Staphylococcus aureus* by Novel Depsipeptides from a Marine *Photobacterium*

**DOI:** 10.3390/md9122537

**Published:** 2011-12-07

**Authors:** Maria Mansson, Anita Nielsen, Louise Kjærulff, Charlotte H. Gotfredsen, Matthias Wietz, Hanne Ingmer, Lone Gram, Thomas O. Larsen

**Affiliations:** 1 Center for Microbial Biotechnology, Department of Systems Biology, Technical University of Denmark, DK-2800 Kgs. Lyngby, Denmark; Email: tol@bio.dtu.dk; 2 Department of Veterinary Disease Biology, Faculty of Life Sciences, University of Copenhagen, DK-1870 Frederiksberg C, Denmark; Email: anini@life.ku.dk (A.N.); hi@life.ku.dk (H.I.); 3 Department of Chemistry, Technical University of Denmark, DK-2800 Kgs. Lyngby, Denmark; Email: lokja@kemi.dtu.dk (L.K.); chg@kemi.dtu.dk (C.H.G.); 4 National Food Institute, Technical University of Denmark, DK-2800 Kgs. Lyngby, Denmark; Email: mwie@food.dtu.dk (M.W.); gram@food.dtu.dk (L.G.)

**Keywords:** *Photobacterium*, *Vibrionaceae*, antivirulence, quorum sensing inhibition, *agr*

## Abstract

During a global research expedition, more than five hundred marine bacterial strains capable of inhibiting the growth of pathogenic bacteria were collected. The purpose of the present study was to determine if these marine bacteria are also a source of compounds that interfere with the *agr* quorum sensing system that controls virulence gene expression in *Staphylococcus aureus*. Using a gene reporter fusion bioassay, we recorded *agr* interference as enhanced expression of *spa*, encoding Protein A, concomitantly with reduced expression of *hla*, encoding α-hemolysin, and *rnaIII* encoding RNAIII, the effector molecule of *agr*. A marine *Photobacterium* produced compounds interfering with *agr* in *S. aureus* strain 8325-4, and bioassay-guided fractionation of crude extracts led to the isolation of two novel cyclodepsipeptides, designated solonamide A and B. Northern blot analysis confirmed the *agr* interfering activity of pure solonamides in both *S. aureus* strain 8325-4 and the highly virulent, community-acquired strain USA300 (CA-MRSA). To our knowledge, this is the first report of inhibitors of the *agr* system by a marine bacterium.

## 1. Introduction

Microorganisms are an attractive source of new natural products with antimicrobial properties [1,2], and the marine environment constitutes a prolific resource of bioactive microorganisms [3,4,5]. Many marine microenvironments stimulate the production of specific metabolites as a response to environmental factors [6]. It is likely that some of these metabolites mediate both intra- and interspecies microbial interactions, and can be seen as potential new scaffolds for development of drug lead candidates [6,7]. The increasing problem of antibiotic resistance among human pathogens highlights the need for novel therapeutic strategies [8]. The search for new avenues in microbial control has therefore been extended from traditional bacteriostatic or bacteriolytic compounds to compounds that target, for example, quorum sensing (QS) pathways [9,10,11]. Quorum sensing inhibitors (QSI) do not necessarily kill or inhibit the growth of a pathogen but rather modulate microbial phenotypes, for example by attenuating virulence [12,13]. *In vivo* studies with QS inhibitory compounds demonstrated how these can be used to slow the spread of infection [14] or enhance the clearance of pathogens from infected tissue [10].

*Staphylococcus aureus* is one of the main causes of nosocomial infections, and methicillin-resistant *S. aureus* (MRSA) are emerging at an alarming rate [15,16]. The virulence of *S. aureus* is ascribed to a number of virulence factors, including extracellular toxins such as α-hemolysin encoded by *hla*, and cell surface adhesion factors such as Protein A encoded by *spa* [17]. Their expression is coordinated through several key regulators, of which the *agr* (accessory gene regulator) QS system is central [18]. This system is a classical two-component system with a sensor histidine kinase, AgrC, and a response regulator, AgrA, in addition to AgrB and AgrD which are responsible for the production of the quorum sensing signal [19,20]. *agr*-dependent QS is mediated via autoinducing peptides (AIP), 16-membered thiolactone macrocycles carrying a peptide tail that control virulence gene expression through the effector molecule RNAIII [21,22]. Structure-activity relationship studies (SAR) demonstrated that while the tail of the AIP is essential for *agr* activation [19,23], the macrocyclic ring is responsible for antagonistic activity [23]. This has led to the synthesis of global inhibitors based on truncated AIPs [23,24]; however, there are only few reports of natural antagonists of this system [24,25,26,27]. Nielsen *et al.* [28] recently developed a screening assay based on *S. aureus lacZ* reporter fusion strains, where the effect of compounds or extracts on expression of three key virulence genes (*spa*, *hla*, and *rnaIII*) and hence potential interference with the *agr* locus can be assessed. Subsequently, the assay was used to identify two xanthones as novel quorum sensing interfering compounds in *S. aureus* [28]. 

We recently established a global collection of marine bacteria with antibacterial activity [29]. The purpose of the present study was to determine if this strain collection also harbored organisms that produced other types of bioactive compounds and we screened pure cultures, crude extracts, and purified secondary metabolites from the collection for potential inhibitors of the *agr* system. One of the bacterial families we investigated was the *Vibrionaceae*. These bacteria are ubiquitous in marine and brackish environments and often associated with marine organisms [30]. The *Vibrionaceae* consist of seven genera, with the majority of species belonging to the *Vibrio* and *Photobacterium* genera. *Vibrio* spp. can be pathogenic to humans [31,32,33] or marine animals [30], but also occur in the commensal microflora of zooplankton [30] or live as bioluminescent symbionts with squid or fish [34,35,36]. The *Photobacterium* genus similarly comprises symbiotic [37,38] and pathogenic species [39,40,41]. Members of the *Vibrionaceae* produce broad-range inhibitory compounds [7,29]; however, only few of the antibacterial compounds have been isolated to date [42]. Antimicrobial compounds from *Vibrio* species include the pyrrolidinediones andrimid [43,44,45] and moiramide B [46] that inhibit fatty acid synthesis [47]. In addition, we recently reported the production of the potent pyrrothine antibiotic holomycin by a marine *Photobacterium* [45].

Herein, we report the isolation and chemical investigation of two novel depsipeptides produced by that same *Photobacterium* strain. The compounds, designated solonamides A and B, inhibit the *agr* QS system of *S. aureus* and therefore interfere with its virulence gene expression. This indicates that marine bacteria are a source of novel chemistry with potential use in antibacterial therapy.

## 2. Results and Discussion

### 2.1. Identification of QS Inhibitors from *Photobacterium* sp.

**Figure 1:**
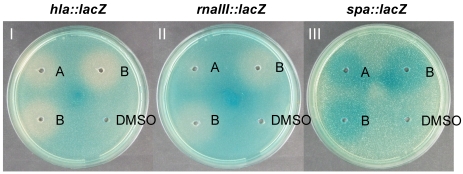
Effect of solonamides (A and B) on *hla*, *rnaIII* and *spa* expression. Solonamides (5 mg mL^−1^) were added to wells in TSA plates containing the 8325-4 derived *lacZ* reporter strains PC322 (*hla::lacZ*), SH101F7 (*rnaIII::lacZ*), or PC203 (*spa::lacZ*). Incubation time was 15 h for plate I and II, and 35 h for plate III (plate numbering indicated with white letters). Solonamide B tested in two wells.

In an initial search for antimicrobial compounds we isolated strain S2753 related to *Photobacterium halotolerans* [29]. Subsequently, the known antibiotic, holomycin, was identified as responsible for its growth inhibitory activity [45]. When investigating ethyl acetate extracts of S2753 in an agar diffusion assay monitoring expression of the *S. aureus* virulence genes *hla*, *rnaIII*, and *spa* [28], we observed an increased expression of *spa* and decreased expression of *hla* and *rnaIII*. The inverse effect of the extracts on *spa* and *hla*/*rnaIII* expression, respectively, indicates the presence of at least one compound that interferes with the *S. aureus* *agr* QS system [28]. Secondary screening of the extract by explorative solid-phase extraction (E-SPE) [48] detected the potential QSI activity in a fraction that did not inhibit growth of *S. aureus* or *V. anguillarum* (data not shown). Bioassay-guided fractionation by diol and C-18 columns resulted in the isolation of two compounds active in the *S. aureus* agar diffusion assay (Figure 1). The activity of the pure compounds matched the initial activity of the extract, confirming that these compounds are responsible for the observed changes in gene expression.

### 2.2. Structural Elucidation of the Solonamides

**Figure 2:**
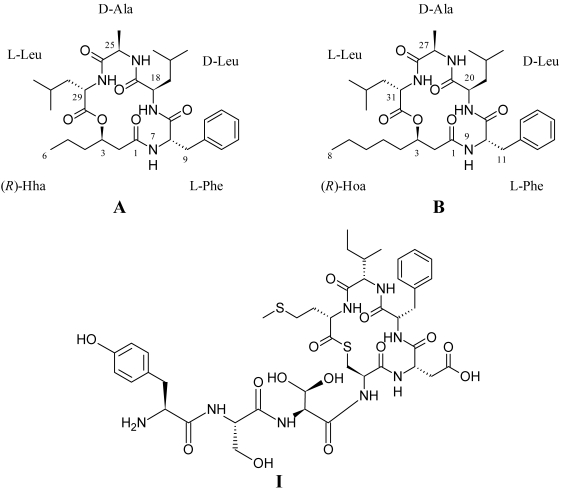
Structures of solonamides A and B produced by *Photobacterium* sp. strain S2753 and structure of natural group I AIP [21].

The solonamides were isolated as white powder with respective molecular formulas C_30_H_46_N_4_O_6_ (A) and C_32_H_50_N_4_O_6_ (B) as determined by HRMS (1 ppm mass accuracy). Analysis of NMR data characterized the structures of the solonamides as cyclodepsipeptides consisting of four amino acids and a 3-hydroxy fatty acid (Figure 2). The amino acid composition was elucidated as alanine, phenylalanine, and two leucines for both peptides based on DQF-COSY and HSQC NMR data.

The spin systems of the amino acids were confirmed through strong and unambiguous H2BC correlations [49] and the carbonyl signals assigned by HMBC correlations. Through careful inspection of the DQF-COSY and H2BC NMR data, solonamide A was found to contain a 3-hydroxyhexanoic acid (Hha), while solonamide B contained a 3-hydroxyoctanoic acid (Hoa). Long-range HMBC and NOESY correlation data allowed the sequence of amino acids to be established as fatty acid-Phe-Leu-Ala-Leu (Figure in Supplementary Information). This was corroborated by MS-MS experiments giving the exact molecular formula of the fragments (2 ppm mass accuracy, see Supplementary Information). The signal from one oxygen-bearing carbon with a high carbon shift indicated an ester linkage. The ring closure linkage was secured by HMBC correlations from H-3 in the fatty acid moiety to the carbonyl in Leu and a weak NOESY correlation from H-3 to the Leu amide and Hα protons. In total, this accounted for the ten degrees of unsaturation resulting from the macrocyclic ring, five carbonyls, and the phenyl group.

The absolute configurations of the individual amino acids were established by acid hydrolysis and Marfey’s method with UHPLC analysis. Both peptides were found to contain L-Phe, D-Ala, and an enantiomeric pair of L-Leu and D-Leu. Acid hydrolysis of the reduced linear peptides and subsequent Marfey’s derivatization specified the stereochemistry of the two Leu, exchanging the L-Leu peak (RT 3.77 min) with a new peak (RT 3.73 min), attributable to the corresponding alcohol. Thus, L- and D-stereochemistry was assigned as fatty acid-L-Phe-D-Leu-D-Ala-L-Leu in both solonamide A and B. 

**Figure 3:**
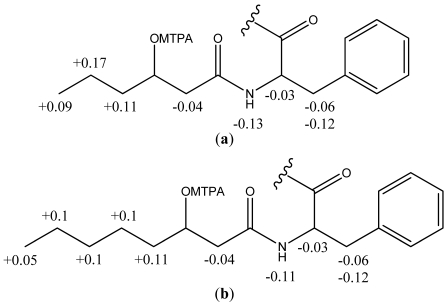
Distribution of the Δδ values (ppm) calculated for the (**a**) 3-hydroxyhexanoic acid (Hha) and (**b**) 3-hydroxyoctanoic acid (Hoa) in the (*R*)- and (*S*)-Mosher’s esters.

The absolute configuration of the fatty acid was established by NMR spectroscopic analysis of the ^1^H and ^19^F chemical shift differences (Δδ*^SR^*) in the (*R*)- and (*S*)-Mosher’s esters analysis of solonamide A and B. The absolute stereochemistry of C-3 in the 3-hydroxy fatty acid was established as (*R*) in both depsipeptides (Figure 3).

The yield of solonamides was up to 10 mg L^−1^, which is a high organic yield compared to that of other γ-proteobacteria [50,51]. This suggests that they could be storage compounds accumulated during growth. However, the solonamides were also produced on a chitin based minimal medium (Supplementary Information) which indicated that these compounds may be produced in the natural habitat of vibrios, such as chitinous zooplankton. In addition, D-alanine and L-leucine are incorporated in the structure; amino acids that are not present in the laboratory medium. Thus, the *Photobacterium* seems to produce these specific stereoforms rather than incorporating the available amino acids. 

### 2.3. Production of Solonamides by Related *Photobacterium* Strains

To test whether the solonamides are also produced by strains related to our *Photobacterium* isolate, we compared strain S2753 with *P. halotolerans* LMG 22194^T^, *P. rosenbergii* LMG 22223^T^, and *P. angustum* S14 [52]. None of these strains produced solonamides (as confirmed by LC-UV/MS), and none affected virulence gene expression in the gene-reporter agar diffusion assay. None of the three strains inhibited growth of *V. anguillarum*, and holomycin, the antibiotic of S2753 [45], was not detected. 

To the best of our knowledge, only two species of *Photobacterium* have been investigated for their secondary metabolites so far [51,53,54]. Oku *et al*. [51] isolated unnarmicin A and C from a marine *Photobacterium* strain MBIC06485 related to *P. leiognathi*. Like the solonamides, unnarmicin A and C consist of four amino acids (L-Phe, L-Leu, D-Phe, L-Leu) and a 3-hydroxyoctanoic and 3-hydroxyhexanoic fatty acid, respectively. The finding of the unnarmicins in another marine *Photobacterium* sp. indicates that production of such peptides could be a common feature in this group of bacteria, despite the absence of solonamides in any related strains that we investigated. 

### 2.4. Solonamides Interfere with *agr*

**Figure 4:**
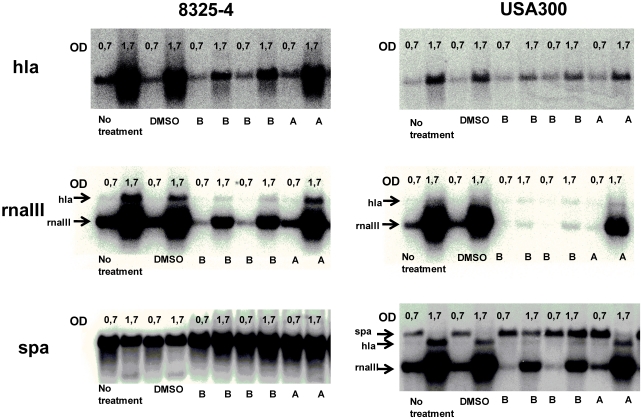
Effect of solonamide A and B on virulence gene expression in *S. aureus* strain 8325-4 [18] and USA300 [55] examined by Northern blot analysis. Solonamides were added to exponentially growing cultures at OD_600_ = 0.4, and RNA was purified at OD_600_ = 0.7 and 1.7. The RNA was reacted with probes recognizing *hla*, *rnaIII*, and *spa*, respectively. Solonamide B tested in duplicates. DMSO was used as negative control.

To verify that the purified solonamides do in fact cause transcriptional changes in virulence gene expression and to assess if the effect is strain specific, we isolated mRNA from *S. aureus* 8325-4 and the community-acquired strain, USA300 at different stages of growth following solonamide exposure and monitored gene expression by Northern blot analysis. Solonamide B dramatically reduced the expression of both *hla* and *rnaIII* while increasing expression of *spa*, strongly indicating that the compound interferes with *agr* regulation (Figure 4). The decreased expression of *rnaIII* was even more pronounced in the highly virulent USA300 strain where high *agr* activity is suspected to be a main contributor to the virulence of the strain [55,56]. The solonamides did not affect the growth rate of the liquid *S. aureus* cultures. Solonamide A was able to increase *spa* expression, but caused only a marginal reduction of *hla* and *rnaIII* expression in both 8325-4 and USA300. The discrepancy between the Northern blot analysis and the agar diffusion assay (mainly with regard to *hla*) may be rooted in the much higher concentrations of solonamides that are used in the agar diffusion assay as compared to the Northern blot analysis. Also, the Northern blot analysis directly monitors the amount of mRNA shortly after solonamide addition, whereas the agar diffusion assay relies on the accumulation of β-galactosidase enzyme over a period of 15 or 35 h. 

The structural similarity of the solonamides and the AIPs (Figure 2) suggest that they may be competitive inhibitors of the *agr* system. Unlike the AIPs, the solonamides are cyclized through a 3-hydroxy fatty acid forming a lactone rather than a thiolactone. However, synthetic lactone and lactam variations of natural AIPs have been found to have antagonistic activity [19,23,57], which our study corroborates. While inhibition of *agr* by AIPs is more tolerant of sequence and structural diversity than is activation [23], Mayville *et al*. [14] found that the presence of the hydrophobic leucine and phenylalanine residues is important for the inhibition of the *agr* response. Both solonamides contain a leucine and phenylalanine; however, the reduced activity of solonamide A indicates that the overall hydrophobicity of the depsipeptides affected by the varying length of the fatty acid moiety might be an important factor influencing activity. 

The solonamides are the first reported antagonists produced by a natural source with a structure resembling that of native *S. aureus* AIPs. Kiran *et al*. (2008) [25] identified hamamelitannin from *Hamamelis virginiana* (witch hazel) as an inhibitor of RNAIII and δ-hemolysin production in *S. aureus* 8325-4, USA300, and clinical *S. epidermidis* isolate MH. Also, ambuic acid from an unidentified fungal strain was found to attenuate *agr* [26].Given the relatively low abundance of staphylococci in the marine environment, it seems unlikely that the *Photobacterium* sp. S2753 produces solonamides as part of a deliberate strategy to interfere with this specific type of bacteria. However, the solonamides might be targeted at other Gram-positive bacteria in the marine environment, such as bacilli and actinobacteria, though little is known about quorum sensing pathways in marine Gram-positive bacteria. A large number of different QS systems have been characterized from Gram-negative bacteria in the marine environment [57,58,59,60,61,62]. We speculate that the solonamides could also affect such systems despite sharing little structural similarity to agonists and antagonists of the systems identified to date [63]. Acylated homoserine lactones, the most widely researched type of QS molecules in Gram-negatives, can serve as both agonists and antagonists in different systems [27], and thus the solonamides may also serve as quorum sensing signals for the *Photobacterium* itself. However, we did not detect solonamides or compounds with similar QSI activity in any of the related strains.

Our findings suggest that quorum sensing inhibition could be an alternative therapeutic strategy for treatment of MRSA *S. aureus* infections; however, the effect in an *in vivo* infection model needs to be tested. Many genes are under QS control both in Gram-positive and Gram-negative bacteria, and thus it is not a simple drug target [64,65]. For example, biofilm formation in *S. aureus* has been linked to low QS activity [66], and so there is a risk that the use of QS inhibitors could lead to decreased susceptibility of traditional antibiotics. Also, it is still unknown how QS inhibition will affect the overall fitness of a pathogen under *in vivo* conditions and thus pose a selective pressure for development of resistance [67] or enhanced virulence [68,69].

## 3. Experimental Section

### 3.1. Isolation and Identification of Strain S2753

Bacterial strain S2753 was isolated from a mussel surface collected in the tropical Pacific (9.1°S 156.8°E) during the Danish Galathea 3 expedition [29]. S2753 was assigned to the *Vibrionaceae* by 16S rRNA gene sequence similarity [29] and identified as being closely related to *Photobacterium halotolerans* based on *recA* and *rpoA* gene sequences, with homologies of 87% (*recA*) and 94% (*rpoA*) [45]. BLAST analyses showed that other closely related species were *Photobacterium rosenbergii* and *Photobacterium angustum* S14 [52]. 

### 3.2. Initial Screening for Anti-Virulence Compounds

S2753 was cultured in each 30 mL of (i) marine minimal medium (MMM) [70] containing 0.4% glucose and 0.3% casamino acids; (ii) Marine Broth 2216 (MB; Difco 2216); (iii) Sigma Sea Salts (SSS; Sigma S9883; 40 g L^−1^) containing 0.4% glucose and 0.3% casamino acids, and (iv) MMM containing 0.2% colloidal chitin [71,72] to investigate the best conditions for production of antibacterial compounds. All cultures were incubated aerated (200 rpm) for 72 h at 25 °C. All cultures were extracted with an equal volume of ethyl acetate (EtOAc). The extract was evaporated under nitrogen until dryness and redissolved in 300 μL 80% v/v ethanol (EtOH) in water. 20 μL of the extract was tested in an agar diffusion assay where expression from promoters of *hla*, *rnaIII*, and *spa* is monitored [28]. 

For further screening, the culture broth of S2753 grown in MMM (1 L, 72 h, 25 °C, 100 rpm) was extracted directly with sterile Diaion HP20SS (12 g L^−1^, 24 h) (Sigma-Aldrich, St. Louis, MO). The resin was filtered off and washed with 80% (v/v) acetonitrile (MeCN)/water (300 mL) and the extract evaporated until dryness on a rotary evaporator. From this extract, 10 mg dry material was subjected to an explorative solid-phase extraction (E-SPE) protocol [48]. This yielded 15 fractions for re-testing in the agar diffusion assay as described above. The E-SPE fractions were also tested for antibacterial activity against *Vibrio anguillarum* strain 90-11-287 and *S. aureus* 8325 in a well diffusion agar assay [73]. 

### 3.3. Isolation and Structural Elucidation of Solonamide A and B

Using 10 L glass fermentors, S2753 was cultured in 5 × 4 L SSS (iii, above) containing 0.4% glucose and 0.3% casamino acids (25 °C, 72 h, 100 rpm) as this medium gave comparable yields to that of MMM (i) but at a lower cost. The broth was extracted directly with Diaion HP20SS (12 g L^−1^) as described above. The extract (3.4 g) was redissolved in EtOAc, absorbed onto 5 g Isolute diol (Biotage, Uppsala, Sweden), and added to a glass column with pure diol (95 g). A total of 12 fractions were collected from the diol column (100 g, 20 × 350 mm) ranging from heptane, dichlormethane (DCM), EtOAc, to pure methanol (MeOH), running under gravity. The fractions containing the QSI compounds (fraction 5, 20:80 (v/v) EtOAc/DCM and fraction 6, 40:60 (v/v) EtOAc/DCM) were separated on Sepra ZT C_18_ (Phenomenex, Torrance, CA)(10 g SNAP) on an Isolera automated flash system (Biotage) using a MeCN/water gradient 25–75% over 20 min (12 mL min^−1^). Pure compounds were obtained directly: Solonamide A (17 mg) and B (201 mg). Activity of pure compounds was tested in the agar diffusion assay as described above (20 μL per well), with a final concentration of compounds of 5 mg mL^−1^ in dimethyl sulfoxide (DMSO). 

NMR spectra were recorded on a Varian Unity Inova 500 MHz spectrometer equipped with a 5 mm probe using standard pulse sequences. The signals of the solvent were used as internal references (δ_H_ 2.49 and δ_C_ 39.5 ppm for DMSO). Carbonyl shifts were confirmed with ^13^C 1D on a Bruker Avance 800 MHz spectrometer with a 5 mm TCI cryoprobe at the Danish Instrument Center for NMR Spectroscopy of Biological Macromolecules.

LC-MS and LC-MS/MS analyses were performed on a maXis quadrupole time of flight mass spectrometer (Bruker Daltonics, Bremen, Germany) equipped with an electrospray (ESI) ion source. The MS was connected to an Ultimate 3000 UHPLC system (Dionex, Sunnyvale, CA) equipped with a diode-array detector. Separation was performed at 40 °C on a 150 mm × 2.1 mm ID, 2.6 µm Kinetex C_18_ column (Phenomenex) using a linear water/MeCN (both buffered with 20 mM formic acid) gradient starting from 15% MeCN and increased to 100% in 13 min at a flow of 0.4 mL min^−1^. The MS and MS/MS experiments were performed in ESI^+^ with a data acquisition range of *m/z* 100–1200 with collision energy of 27 V. The MS was calibrated using sodium formate automatically infused prior to each analytical run, providing a mass accuracy of below 1 ppm in MS mode and 2 ppm in MS/MS mode. 

The absolute configuration of the amino acids were found using acid hydrolysis (6 M HCl, 110 °C, 20 h) [74] and derivatisation with Marfey’s reagent (1-fluoro-2,4-dinitrophenyl-5-l-alanine amide, FDAA, Sigma-Aldrich) following the protocol by Bonnard *et al*. [75]. Ultra-high liquid chromatography-diode array (UHPLC-UV) analyses of the amino acids were done on a Dionex RSLC Ultimate 3000 (Dionex) equipped with a diode-array detector. Separation was obtained on a Kinetex C_18_ column (150 × 2.10 mm, 2.6 μm, Phenomenex) maintained at 60 °C using a linear gradient starting from 25% MeCN in water (both buffered with 50 ppm TFA) increasing to 27% MeCN over 6 min at a flow rate of 0.8 mL min^−1^. Retention times of the FDAA amino acid derivatives used as standards were as follows (maximum standard deviation ± 0.002 min): FDAA (1.50 min), L-Ala (1.14 min), D-Ala (1.61 min), L-Phe (3.58 min), D-Phe (5.04 min), L-Leu (3.77 min), D-Leu (5.49 min), comparable to the observed retention times from the solonamide-derived amino acids. 

To specify the stereochemistry of enantiomeric amino acids, the depsipeptides were reduced by LiBH_4_. The resulting linear peptides were subjected to the above mentioned acid hydrolysis and Marfey’s derivatisation. Details are given in the Supplementary Information. 

For the absolute configuration of the fatty acid residues, the (*R*)- and (*S*)-Mosher’s esters were prepared for both depsipeptides, and the stereocenters were assigned based on their ^1^H and ^19^F chemical shift differences (Δδ*^SR^*) [76,77]. Details are given in the Supplementary Information. 

*Solonamide A*: white amorphous solids; UV (MeCN/H_2_O) λ_max_ 200 nm (100%); for NMR data refer to Table 1; HRESIMS *m/z* 558.3486 (calcd for C_30_H_46_N_4_O_6_, 558.3496). 

*Solonamide B*: white amorphous solids; UV (MeCN/H_2_O) λ_max_ 200 nm (100%); for NMR data refer to Table 1; HRESIMS *m/z* 586.3725 (calcd for C_32_H_50_N_4_O_6_, 586.3730). 

**Table 1:** NMR spectroscopic data (DMSO-*d*_6_) of solonamide A and B.

	A				B			
	Atom	δ_C_ (ppm)	δ_H_ (ppm)(multiplicity, *J* (Hz))	HMBC	Atom	δ_C_ (ppm)	δ_H_ (ppm)(multiplicity, *J* (Hz))	HMBC
**Hha/Hoa**	1	170.6	-		1	170.4	-	-
	2	40.7	2.67 (1H, dd, 13.5, 3.8) 2.11 (1H, dd, 13.5, 10.1)	1,3,4	2	40.5	2.69 (1H, dd, 13.5, 3.7) 2.11 (1H, dd, 13.5, 10.3)	1,3,4
	3	72.1	5.14 (1H, m)	1,5	3	72.0	5.13 (1H, m)	1,4,5,36
	4	36.2	1.62 (1H, m) 1.45 (1H, m)	2,3,6	4	33.8	1.65 (1H, m) 1.45 (1H, m)	5,6
	5	17.7	~1.22 (1H, m)	3,4	5	23.8	~1.20 (2H, m)	6,7
	6	13.8	0.85 (3H, t, 7)	4,5	6	30.8	~1.22 (2H, m)	ND
					7	21.8	1.25 (2H, m)	ND
					8	13.6	0.84 (3H, m)	6
**L-Phe**	7-NH	-	8.61 (1H, d, 3.2)	1	9-NH	-	8.59 (1H, d, 3.0)	1,10,11,8
	8-CH_α_	55.9	4.26 (1H, ddd, 10, 6.1, 3.2)	9,10,14	10-CH_α_	55.6	4.26 (1H, ddd, 10, 6.1, 3.3)	11,18
	9-CH_β,1_	36.4	2.94 (1H, dd, 13.3, 6.1) 2.72 (1H, dd, 13.3, 10)	8,10,11/15,14	11-CH_β,1_	36.1	2.95 (1H, dd, 13.3, 6.1) 2.73 (1H, dd, 13.3, 10)	10,12,13/17,18
	10	135.8	-	-	12	136.0	-	-
	11,15	129.1	7.18 (2H, m)	9,13	13,17	128.7	7.20 (2H, m)	11,15
	12,14	128.4	7.25 (2H, m)	10	14,16	128.0	7.26 (2H, m)	12,14/16
	13	126.5	7.18 (1H, m)	11	15	126.2	7.20 (1H, m)	13/17
	16-CO	174.4	-	-	18-CO	174.2	-	-
**D-Leu**	17-NH	-	8.63 (1H, d, 5.6)	16,18,19	19-NH	-	8.64 (1H, d, 5.6)	18,20,21
	18-CH_α_	53.1	3.56 (1H, m)	ND	20-CH_α_	52.7	3.57 (1H, m)	21,22,25
	19-CH_β,1_	38.9	1.36 (1H, m) 1.29 (1H, m)	18,21,22	21-CH_β,1_	38.6	1.31–1.37 (2H, m)	20,22,24
	20-CH_γ_	23.5	0.97 (1H, m)	21	22-CH_γ_	23.3	0.99 (1H, m)	ND
	21-CH_δ,1_	23.2	0.69 (3H, d, 6.6)	19,20	23-CH_δ,1_	22.9	0.70 (3H, d, 6.6)	21,22,24
	22-CH_δ,1_	20.7	0.52 (3H, d, 6.5)	19,20	24-CH_δ,1_	20.4	0.53 (3H, d, 6.4)	21,23
	23-CO	171.8	-	-	25-CO	171.6	-	-
**D-Ala**	24-NH	-	7.53 (1H, d, 8.9)	27,29	26-NH	-	7.48 (1H, d, 8.9)	25,27,28
	25-CH_α_	48.2	4.18 (1H, m)	26,27	27-CH_α_	47.8	4.18 (1H, m)	28,29
	26-CH_β,1_	16.6	1.32 (3H, d, 7.4)	27	28-CH_β,1_	16.4	1.33 (3H, d, 7.3)	27,29
	27-CO	171.3	-	-	29-CO	170.9	-	-
**L-Leu**	28-NH	-	7.08 (1H, d, 10)	27,29	30-NH	-	7.05 (1H, d, 10)	29,31
	29-CH_α_	49.0	4.46 (1H, dt, 10, 4.4)	30,31,34	31-CH_α_	48.6	4.47 (1H, dt, 10, 4.3)	32,33,29/36
	30-CH_β,1_	39.3	1.63 (1H, m) 1.53 (1H, m)	29,31,33,27/34	32-CH_β,1_	39.0	1.64 (1H, m) 1.52 (1H, m)	31,33,35,29/36
	31-CH_γ_	23.9	1.50 (1H, m)	29,30,33	33-CH_γ_	23.8	1.50 (1H, m)	32,34,35
	32-CH_δ,1_	23.2	0.86 (3H, d, 6.5)	30,31,33	34-CH_δ,1_	23.0	0.86 (3H, d, 6.6)	32,33,35
	33-CH_δ,1_	21.3	0.81 (3H, d, 6.5)	30	35-CH_δ,1_	21.1	0.82 (3H, d, 6.4)	32,33
	34-CO	171.2	-	-	36-CO	170.8	-	-

### 3.4. LC-UV/MS Analyses of Related *Photobacterium* Strains

To investigate the potential production of *agr* inhibitors in related *Photobacterium* strains, the metabolite profile of S2753 was compared by liquid chromatography-diode array/mass spectrometry (LC-UV/MS) to *P. halotolerans* (LMG 22194^T^), *P. rosenbergii* (LMG 22223^T^), and *P. angustum* (S14) described by de Nys*et al*. [52]. All strains were grown in 30 mL MMM containing 0.4% glucose and 0.3% casamino acids (25 °C, 72 h, 200 rpm). Cultures were extracted with an equal volume of EtOAc and evaporated under nitrogen. Residues were redissolved in MeOH for LC-UV/MS analyses and in 80% EtOH for bioassay testing. Inhibition of *agr* was tested as described above. Extracts were also tested against *V. anguillarum* strain 90-11-287 for growth inhibition. LC-UV/MS analyses were performed on an Agilent 1100 liquid chromatograph with a diode array detector (Agilent, Waldbronn, Germany) coupled to an LCT TOF mass spectrometer (Micromass, Manchester, UK) using a Z-spray ESI source. Separation was obtained on a Luna II C_18_ column (50 × 2 mm, 3 μm, Phenomenex) fitted with a security guard system using a linear gradient starting from 15% MeCN in water (both buffered with 20 mM formic acid) increasing to 100% MeCN over 20 min at a flow rate of 0.3 mL min^−1^.

### 3.5. Northern Blot Analysis

Northern blot analysis was performed as described previously [78]. The strains used were *S. aureus* FPR 3757 [55], a CA-MRSA USA300 obtained from ATCC (Boras, Sweden), and 8325-4 [17]. Samples for RNA purification were taken from cultures in Tryptone Soya Broth (TSB, Oxoid, Greve, Denmark) shaking at 185 rpm at 37 °C in a water bath (10 mL culture in 100 mL Erlenmeyer flask). Growth was monitored by measuring optical density at OD_600_. Start inoculum was OD_600_ = 0.03. Solonamides were added at OD_600_ = 0.4. Samples for RNA purification were taken at OD_600_ = 0.7 and 1.7. Probes targeting *rnaIII*, *spa*, and *hla* transcripts were amplified by PCR using primers *hla* forward (5′-GGG TTA GCC TGG CCT TCA GCC-3′), *hla* reverse (5′-GGG TGC CAT ATA CCG GGT TC-3′), *spa* forward (5′-GGG GGT GTA GGT ATT GCA TCT G-3′), *spa* reverse (5′-GGG GCT CCT GAA GGA TCG TC-3′), *rnaIII* forward (5′-GGG GAT CAC AGA GAT GTG ATG-3′), and *rnaIII* reverse (5′-GGG CAT AGC ACT GAG TCC AAG G-3′)(TAG Copenhagen A/S, Denmark). The resulting PCR fragments were 311 bp (*hla*), 719 bp (*spa*), and 316 bp (*rnaIII*), respectively. 

## 4. Conclusions

The rapid, worldwide increase in antibiotic-resistant *S. aureus* [15] has led to an intense search for compounds with potential use in alternative therapeutic strategies [9]. Virulence of *S. aureus* involves a complex set of proteins, with the *agr* QS system controlling expression of several of the virulence genes. The investigation of crude extracts and fractions from a marine *Photobacterium* led to the identification of two novel depsipeptides, solonamides A and B, as potent inhibitors of this system. Interestingly, we found that solonamide B interfered with *agr* not only in *S. aureus* 8325-4 but also in strain USA300, which is a predominant community-acquired MRSA (CA-MRSA) strain in the US [79]. This finding suggests that quorum sensing inhibition could be an option for treatment of *S. aureus* USA300 infections. Future experiments will reveal the extent to which the solonamides are effective in treating *S.* *aureus* infections. In combination with other recent work from our laboratory [42,45,71] the present study underlines that vibrios are a promising potential source of novel bioactive secondary metabolites. 

## Supplementary Files

Supplementary File 1: PDF-Document (PDF, 528 KB)
