# Supplementary material for: Inhibition of Virulence Gene Expression in Staphylococcus aureus by Novel Depsipeptides from a Marine Photobacterium
Source: Mar Drugs. 2011 Dec 7;9(12):2537–52. doi: 10.3390/md9122537 (PMC3280567; doi:10.3390/md9122537)
Supplement: Supplementary File 1: — PDF-Document (PDF, 528 KB) [file marinedrugs-09-02537-s001.pdf]

Supplementary Information

A1: LC-MS selective ion-trace of solonamide A (*m/z* 558, RT = 11.43 min) and solonamide B (*m/z* 586, RT = 13.25 min) production on different growth media, including marine minimal media with glucose (MMM), marine broth (MB), sigma sea salts (SSS), and marine minimal media with chitin (Chitin). Slight shift in retention times due to inter-batch variations on the LC-MS system.

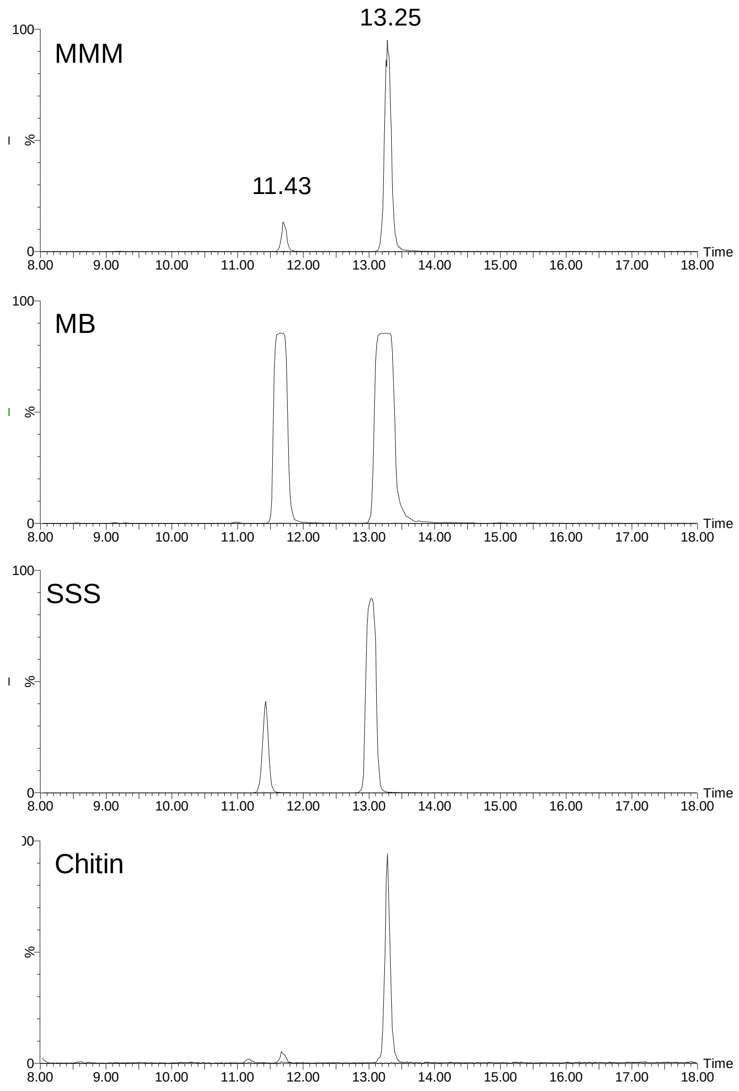

NMR

Assignments of the depsipeptides were performed using a conventional assignment approach. HMBC correlations between 3-H/1-CO, 3-H/36-CO and NOEs between 3-H/31-Hα-Leu confirms the lactone closure of the structure. The figure (**A3**) below shows key correlation for the lactone ring closure.

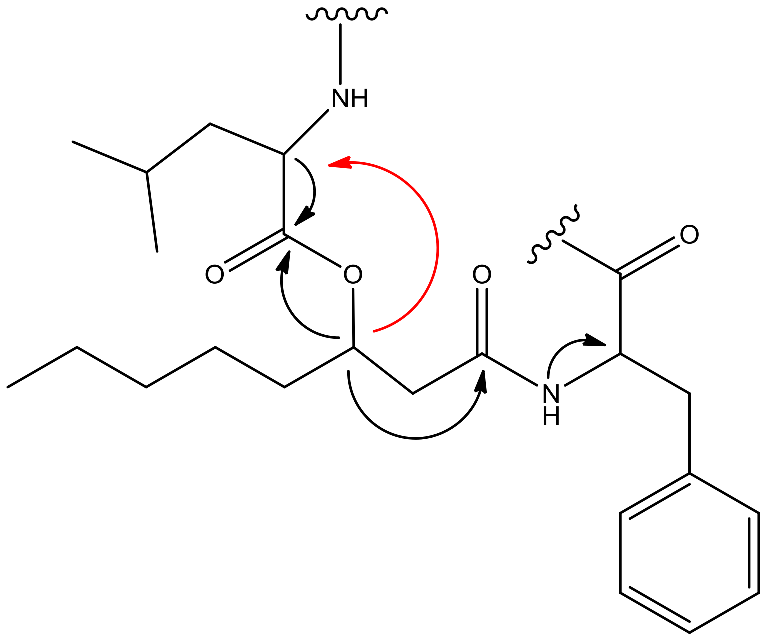

A4. 1D ^{1}H spectrum of Solonamide A

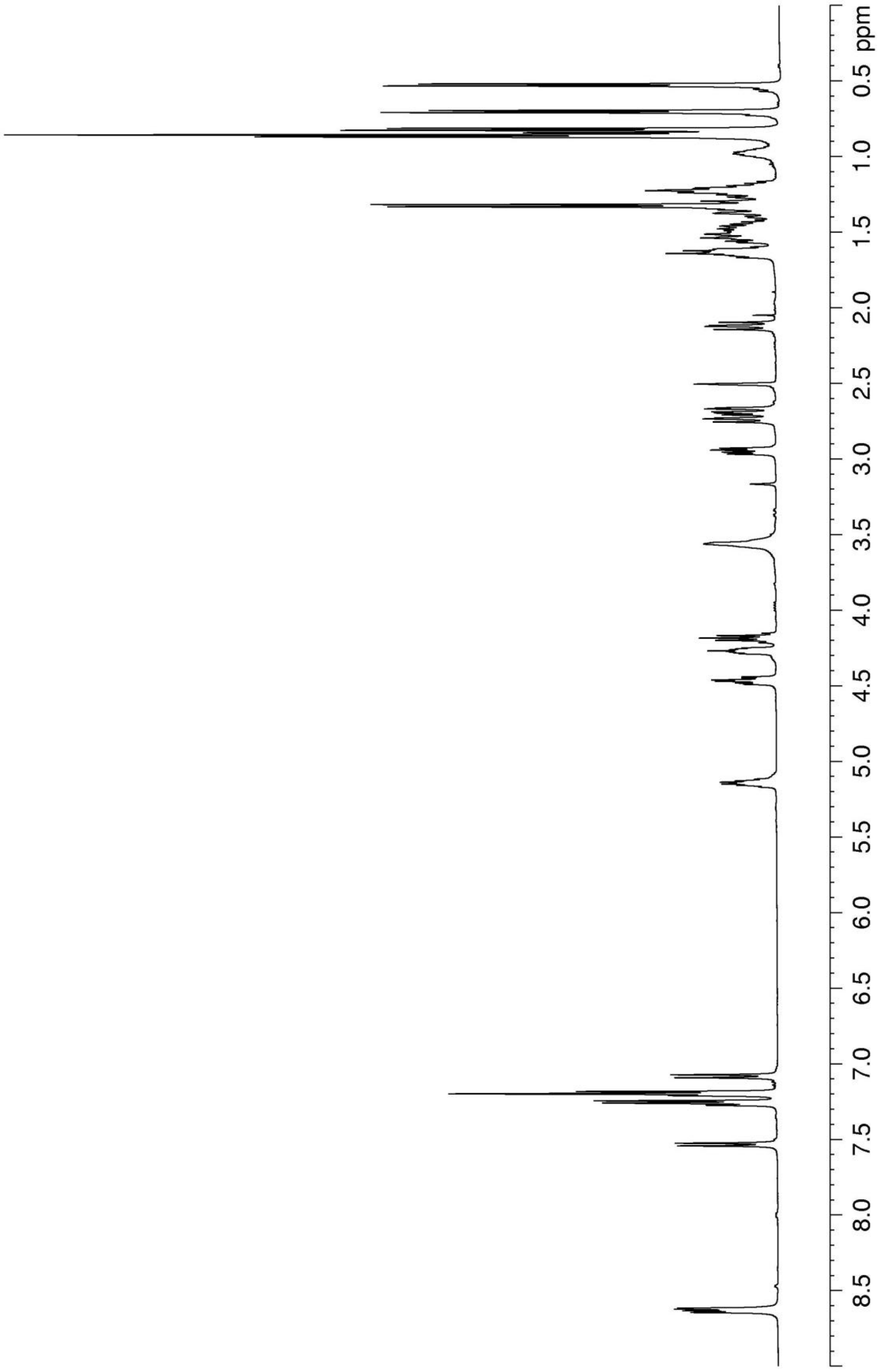

A5. 1D ^{1}H spectrum of Solonamide B

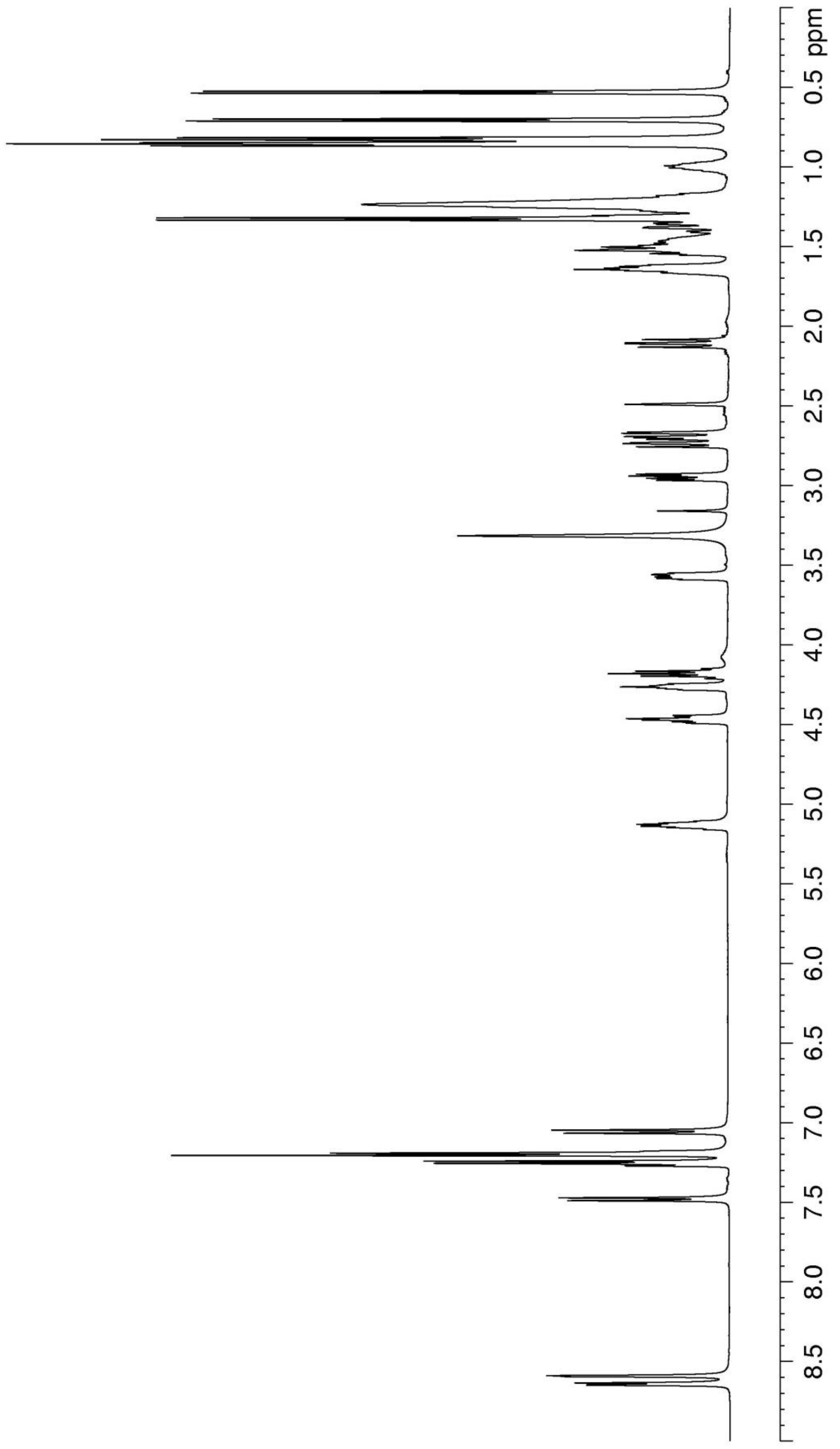

MS/MS data

A6: MS and MS/MS spectrum of solonamide A ($C_{30}H_{46}N_4O_6$, calcd. 558.3496, Δ *m/z* 0.8 ppm) with characteristic fragments and their molecular formulas (Δ *m/z* 0.2-0.6 ppm):

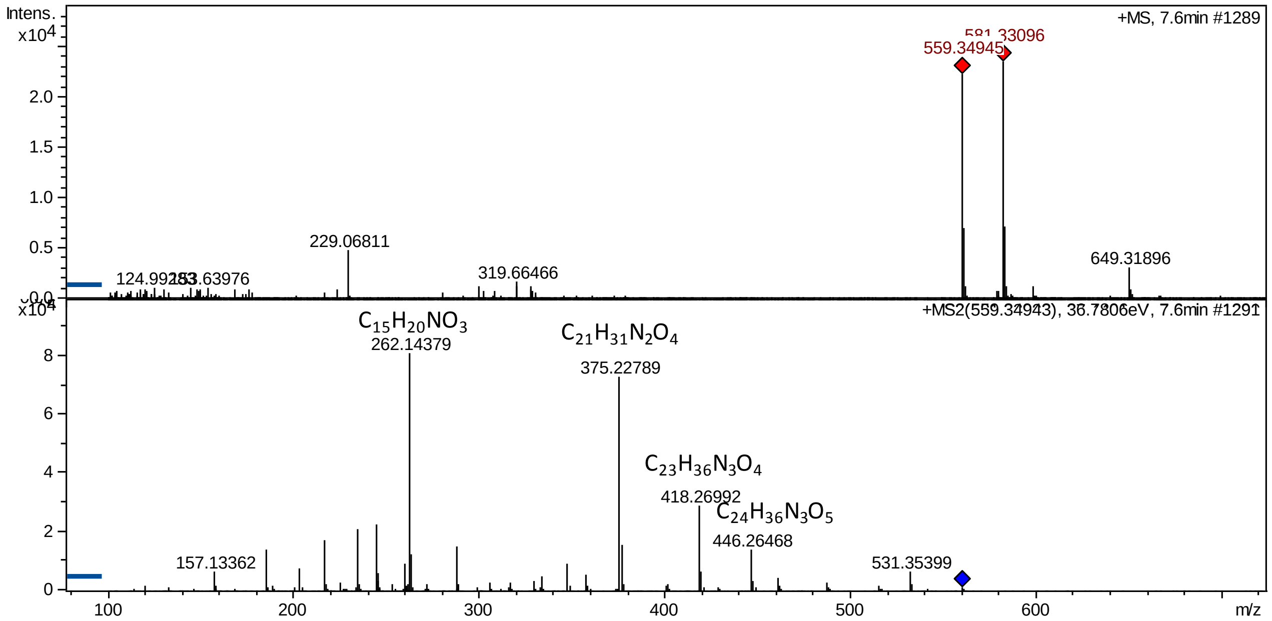

A8: MS and MS/MS spectrum of solonamides B ($C_{32}H_{50}N_4O_6$, calcd. 586.3730, Δ *m/z* 0.0 ppm) with characteristic fragments and their molecular formulas (Δ *m/z* 0.2-0.6 ppm):

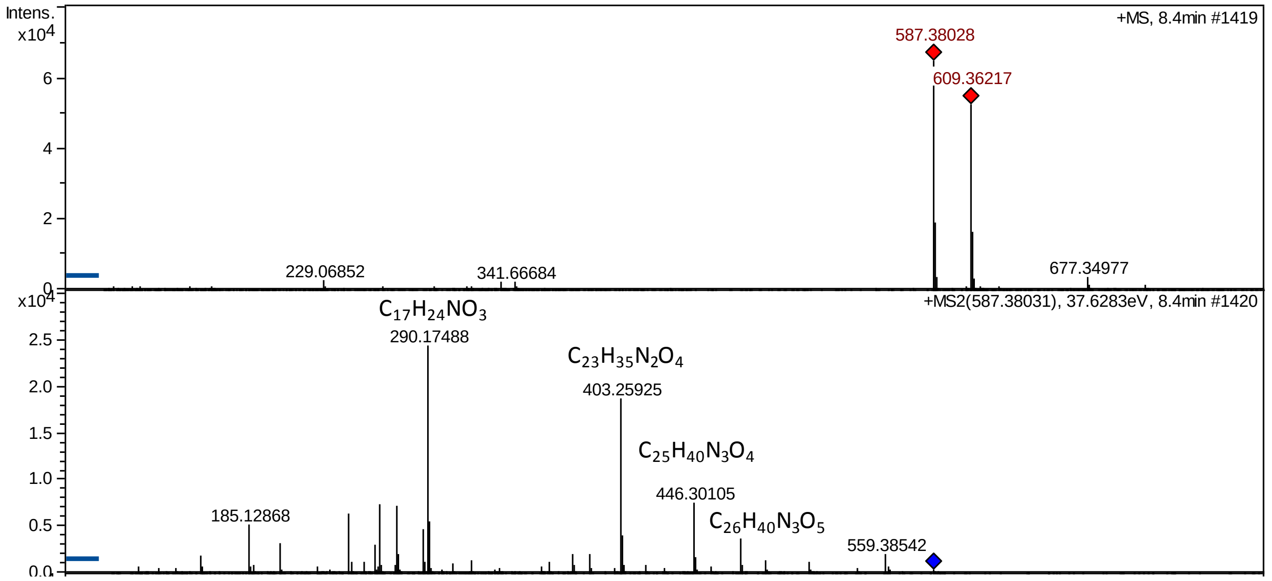

A7: Characteristic fragments for solonamide A (left) and B (right):

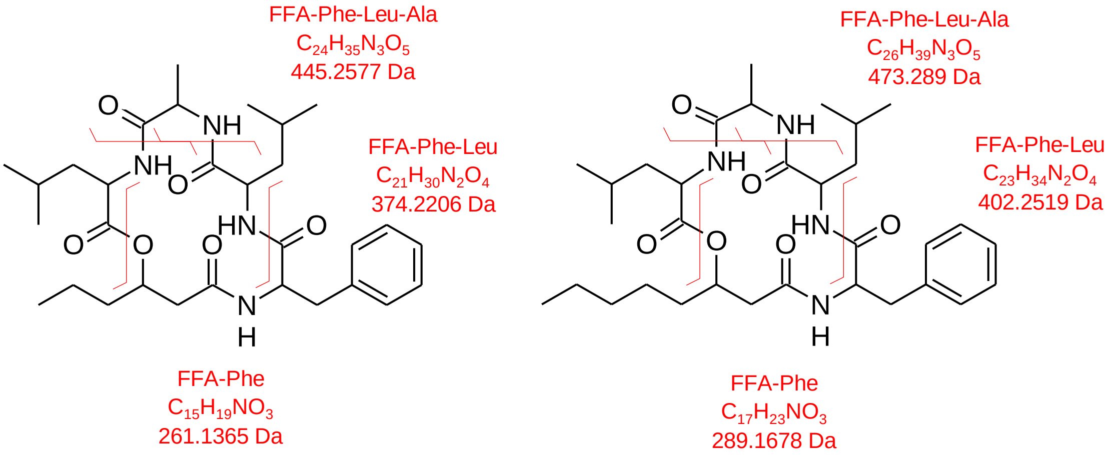

Establishment of absolute configuration

The absolute configuration of C3 at 3-hydroxyhexanoic acid (Hha) and 3-hydroxyoctanoic acid (Hoa) was determined using the Mosher's method. Here with the Mosher's method two enantiomers of an acid chloride (α-methoxy-α-trifluoromethylphenylacetic acid chloride, MTPA-Cl) are reacted with the secondary alcohol at the stereogenic center in order to reveal the configuration of this center. The reaction yields two diastereoisomers with known configuration around one of the stereogenic centers enabling the determination of the other stereogenic center.

The sign of the $\Delta\delta$ values can be seen in the figures below, all pointing toward the same configuration at the C3 stereocenter in Hha and Hoa. The values for $\Delta\delta$ are positive for the alkane end of Hha/Hoa and negative towards the carbonyl end, which points towards an *R*-configration of the C3 stereocenter. The difference seen in the flourine NMR data supports this conclusion when

the alkane chain of the molecule is regarded as least bulky compared to the remaining part of the molecule with a Δδ(SR) ~ -0.68 ppm .

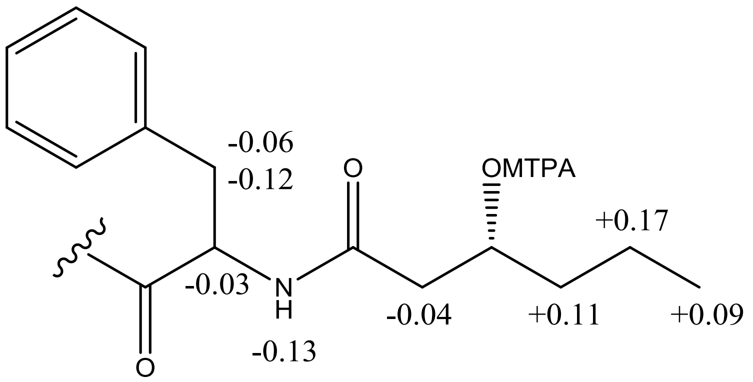

A8. Distribution of the Δδ values calculated for the 3-hydroxyhexanoic acid (Hha) of solonamide A

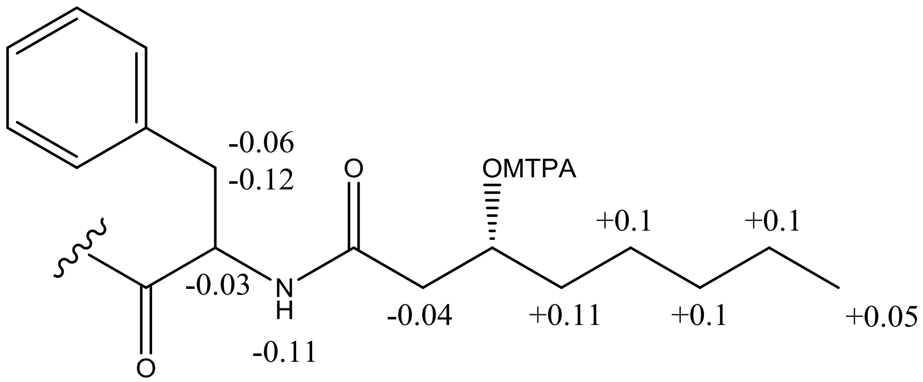

A9. Distribution of the Δδ values calculated for the 3-hydroxyoctanoic acid (Hoa) of solonamide B

Experimental procedure used for Mosher's:

Methanolysis of solonamide A (and B)

5 mg (8.52 μmol) of the depsipeptide (solonamide A) is dissolved in 1.5 mL of 0.5 M NaOMe in MeOH and stirred at room temperature for 1 h. The reaction mixture is neutralised by careful addition of 1M HCl (aq) and the pH is monitored with universal indicator paper. The reaction mixture is concentrated to dryness on a rotary evaporator, and partitioned in EtOAc and H_2O.

Methanolysis of solonamide B was performed using the same procedure. The organic phase is concentrated to dryness on a rotary evaporator and the methyl ester product verified by LC-MS ($[M+H]^+$ A: 591.3803 Da, B: 619.4053 Da) and purified by RP-HPLC on a Waters HPLC with a 600 controller and a 996 photodiode array detector using a water/MeCN gradient elution from 40 to 60 % MeCN over 20 min (5 mL/min) with a Luna C_{18} column (5 µm, 250x10 mm, Phenomenex). Pure methyl esters of solonamide A (1.1 mg) and B (2.7 mg).

Preparation of the *R*-MTPA ester of solonamide A (and B)

To a stirred solution of the purified methanolysis product (0.55 mg, 0.889 µmol) in dry pyridine (150 µL) is added 5 µL (catalytic amount, 0.1 eq) of a solution of dimethylaminopyridine (DMAP) in dry pyridine (0.4 mg, 50 µL). After 10 min 9.1 µL *S*-(+)-MTPA-Cl (48.6 µmol) is added and the reaction is left overnight at room temperature. The reaction mixture is dried on a speedvac, redissolved in MeOH and purified on a Waters HPLC with a 600 controller and a 996 photodiode array detector using a water/MeCN gradient elution from 45 to 100 % MeCN over 20 min (5 mL/min) with a Luna C_{18} (5 µm, 250x10 mm, Phenomenex) column to give pure *R*-MTPA ester (A: 0.5 mg, B: ~1.2 mg).

Preparation of the *S*-MTPA ester of solonamide A (and B)

Using the same procedure as for the *R*-MTPA ester, the *S*-MTPA ester is produced by reaction with *R*-(–)-MTPA-Cl. Pure *R*-MTPA esters (A: 0.4 mg, B: ~1.1 mg).

Establishing stereochemistry of enantiomeric Leu by reduction and Marfey's

2 mg of solonamide B is reduced in 2 M $LiBH_4$ in THF at 0°C for 30 min, then at rt overnight and lastly heated to 50˚C for 30 min. EtOAc is added and the reaction is quenched with sat. NH_4Cl (aq) before the product is extracted with EtOAc, CH_2Cl_2 and Et_2O concentrated and purified by RP-HPLC on a Waters HPLC with a 600 controller and a 996 photodiode array detector using a water/MeCN gradient elution from 25 to 80% MeCN over 20 min (5 mL/min) with a Luna C_{18} column (5 µm, 250x10 mm, Phenomenex). Yields 0.7 mg of reduced open-chain solonamide B. The same procedure is used for solonamide A, only with a gradient of 35-70 %, yielding 0.8 mg.

300 µg of each peptide is hydrolysed with 300 µL 6 M HCl at 110°C for 20 h. To the hydrolysis products is added 75 µL water, 20 µL 1 M $NaHCO_3$ solution and 100 µL 1% FDAA in acetone, followed by reaction at 40°C in 1 h. The vial is removed from the heat, neutralised with 10 µL 2 M HCl and the solution is diluted with 395 µL MeOH to a total volume of 0.5 mL. The FDAA derivatives are analysed by UPLC on a Dionex Ultimate 3000 with a diode array detector and a Kinetex C_{18} column (2.6 µm, 150x2.10 mm, Phenomenex). The analyses are run with a gradient elution of water/MeCN from 25 to 37 % MeCN over 6 min (60°C, 0.8 mL/min) and the FDAA derivatives of the hydrolysates are compared to retention times of the standard amino acid derivatives: D-Ala (1.61 min), L-Ala (1.14 min), D-Leu (5.49 min), L-Leu (3.77 min), D-Phe (5.04 min), L-Phe (3.585 min). FDAA elutes at 1.50 min.
